# Baeyer-Villiger Oxidation of Some C_19_ Steroids by *Penicillium lanosocoeruleum*

**DOI:** 10.3390/molecules181113812

**Published:** 2013-11-07

**Authors:** Alina Świzdor

**Affiliations:** Department of Chemistry, Wroclaw University of Environmental and Life Sciences, Norwida 25, Wroclaw 50-375, Poland; E-Mail: alina.swizdor@up.wroc.pl; Tel.: +48-71-3205-252; Fax: +48-71-3284-124

**Keywords:** steroidal lactones, biotransformation, Baeyer-Villiger oxidation, DHEA, androsterone, epiandrosterone, 5α-androstan-3,17-dione, 3β-HSD, *Penicillium lanosocoeruleum*

## Abstract

The biotransformation of androsterone (**1**), epiandrosterone (**2**), androstanedione (**3**) and DHEA (dehydroepiandrosterone) (**4**) by *Penicillium lanosocoeruleum*—a fungal species not used in biotransformations so far—were described. All the substrates were converted in high yield (70%–99%) into d ring δ-lactones. The oxidation of **1** produced 3α-hydroxy-17a-oxa-d-homo-5α-androstan-17-one (**5**). The oxidation of **2** led to 3β-hydroxy-17a-oxa-d-homo-5α-androstan-17-one (**6**). The biotransformation of **3** resulted in the formation of 3α-hydroxy-17a-oxa-d-homo-5α-androstan-17-one (**5**) and 17a-oxa-d-homo-5α-androstan-3,17-dione (**7**). An analysis of the transformation progress of the studied substrates as a function of time indicates that the Baeyer-Villiger monooxygenase of this fungus does not accept the 3β-hydroxy-5-ene functionality of steroids. In this microorganism steroidal 3β-hydroxy-dehydrogenase (3β-HSD) was active, and as a result DHEA (**4**) was transformed exclusively to testololactone (**8**). Apart from the observed oxidative transformations, a reductive pathway was revealed with the C-3 ketone being reduced to a C-3α-alcohol. It is demonstrated for the first time that the reduction of the 3-keto group of the steroid nucleus can occur in the presence of a ring-D lactone functionality.

## 1. Introduction

Steroids represent an important class of natural products with varying pharmacological properties. Minor modifications in their structure may affect their bioactivity, consequently, research on the preparation of potentially useful steroid analogues is being continued for development of processes exploitable by the pharmaceutical industry. Biotransformations are powerful tools for generation of novel steroidal drugs, as well as for efficient production of steroidal key intermediates. In the majority of cases, these modifications have been achieved by the use of whole-cells microorganisms. The use of enzymes ensures the high regio- and stereoselectivity of the reaction to be performed, and biotransformations are more environmentally friendly in comparison with chemical syntheses. Microbial transformations of various steroids have been reviewed recently [1,2].

Currently research efforts in production of steroids of pharmaceutical interest are focused on either optimization of existing processes or identification of novel potentially useful bioconversions. It is not surprising that several microorganisms have been reported to exhibit Baeyer-Villiger oxidation properties. Only in a few cases the isolated enzymes have been used [1,3,4]. Recently, cyclopentadecanone monooxygenase (CPDMO) isolated from *Pseudomonas* sp., involved in the catabolism of cyclopentadecanone in Nature, was also found to accept steroids as substrates [3]. It has been screened for the Baeyer-Villiger oxidation of a large number of steroids but the products were formed in low yields. The use of the chimeric enzyme PASTMO (a hybrid of phenylacetone monooxygenase (PAMO) and bacterial steroid monooxygenase (STMO) from *Rhodococcus rhodochrous*) in the biotransformation of progesterone afforded the final lactone (testololactone) with a moderate (19%) conversion [4]. Among microorganisms, fungal species of the *Penicillium* [5,6,7,8,9,10], *Aspergillus* [11,12,13,14,15,16,17,18,19,20] and *Fusarium* genera [21,22,23] are especially known to transform 4-en-3-oxo and 3β-hydroxy-5-ene C_21_ as well as C_19_ steroids. Enzymes performing oxidation belong to the family of NADPH-dependent steroid Baeyer-Villiger monooxygenases (BVMOs) containing FAD as a cofactor. The catalytic activity of this class of enzymes is manifested in the transformation of C-20 ketones into the corresponding acetates, which then hydrolyze to the related C-17 alcohols, followed by oxidation to ketones, and finally ring-D Baeyer-Villiger oxidation gives lactones. 

Steroidal lactones are important compounds due to their biological activity such as anticancer and antiandrogenic action. It was found that they could inhibit the 5α-reductase, and as a result of this, block the conversion of testosterone to 5α-dihydrotestosterone [24] and therefore have therapeutic potential for the treatment of androgen-dependent diseases. Steroidal testolactone as an inhibitor of steroidal aromatase [25], is used in the clinical treatment of breast cancer [25] and a few forms of precocious puberty [26]. Although chemical synthesis routes to steroidal ring-D lactones using peracids [27,28] are possible, they often result in a larger variety of products (e.g., regioisomeric lactones, epoxylactones or epoxyketones).

Microbial steroid BVMOs are steroid-induced enzymes, which utilize atmospheric oxygen as oxidant. Their catalytic properties exhibit differences [1], even within the same species of microorganism. For example, *Penicillium citreo-viride* oxidized androstenedione, progesterone and DHEA (dehydroepiandrosterone) to testololactone, while pregnenolone was not transformed; conversion of DHEA to testololactone occurred through the 3β-hydroxysteroid dehydrogenase/5-ene-4-ene isomerase pathway resulting in the generation of a 4-en-3-oxo system in the steroid ring-A [7]. This same pathway was observed during the transformation of DHEA by *P. griseopurpureum* and *P. glabrum* [8]. In contrast to *P. citreo-viride*, BVMO(s) from *P. lilacinum* accepted 3β-hydroxy-5-ene substrates [6]. The enzyme was able to carry out the degradation of 17β-acetyl side chain and ring-D lactonization of 3β-hydroxy-5-ene as well as 4-en-3-oxo substrates with preference for lactonization of 5-ene steroids. In this way DHEA was converted only to 3β-hydroxy-17a-oxa-d-homo-androst-5-en-17-one (a d-lactone with conserved ring-A moiety), whereas pregnenolone was also transformed to testololactone (a d-lactone with a 3-oxo-4-ene system). *P. camemberti* was able to carry out the oxidation of pregnenolone and DHEA to testololactone, which could be formed via two routes: through 4-en-3-ketone or 3β-hydroxylactone [9]. In most cases of transformations described in literature, the steroids, besides the Baeyer-Villiger oxidation, also underwent hydroxylation, reduction or dehydrogenation [8,12,15,16,19,23,29]. The mixtures of metabolites formed during the transformations led to difficulties in product purification. The processes were usually slow (2–5 days) and the productivity was low.

In the light of our interest [6,9,29] in the microbial Baeyer-Villiger oxidation of steroids, this article reports the results of the transformation of a series of androstanes, namely DHEA, epiandrosterone, androsterone and androstanedione, by *Penicillium lanosocoeruleum* KCH 3012. To the best of the author’s knowledge, this is the first report of the use of *P. lanosocoeruleum* in biotransformations.

## 2. Results and Discussion

**Scheme 1:**
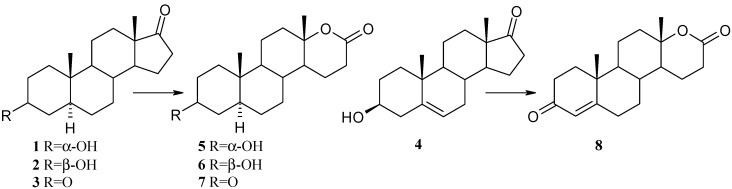
Structures of substrates and products of their transformations.

The *P. lanosocoeruleum* KCH 3012 strain produces a Baeyer-Villiger monooxygenase which is able to carry out the regioselective ring-D lactonization of 5α-dihydro-steroids (**1**–**3**) as well as unsaturated steroid **4** (Scheme 1, Table 1). The corresponding lactones **5** and **6**, were formed with high yield (70%–96%) as the sole products from epimeric 3-hydroxy substrates—androsterone (**1**) and epiandrosterone (**2**). Two lactones: 3α-hydroxy-17a-oxa-d-homo-5α-androstan-17-one (**5**) and 17a-oxa-d-homo-5α-androstan-3,17-dione (**7**) were isolated after transformation of a 3,17-diketo compound—androstanedione (**3**). DHEA (**4**) was converted to testololactone (**8**).

**Table 1:** The results of transformation of **1**–**4** and **7** by *P. lanosocoeruleum*.

Starting material	Time (h)	Compounds	Yield (%) ^a,b^
Androsterone (**1**)	48	Androsterone (**1**)	-
		3α-Hydroxy-17a-oxa-d-homo-5α-androstan-17-one (**5**)	98 (96)
Epiandrosterone (**2**)	48	Epiandrosterone (**2**)	19 (17)
		3β-Hydroxy-17a-oxa-d-homo-5α-androstan-17-one (**6**)	75 (70)
Androstanedione (**3**)	24	Androstanedione (**3**)	-
		17a-Oxa-d-homo-5α-androstan-3,17-dione (**7**)	40 (37)
		3α-Hydroxy-17a-oxa-d-homo-5α-androstan-17-one (**5**)	59 (55)
DHEA (**4**)	24	DHEA (**4**)	-
		Testololactone (**8**)	96 (91)
17a-Oxa-d-homo-5α-androstan-3,17-dione (**7**)	48	17a-Oxa-d-homo-5α-androstan-3,17-dione (**7**) 3α-Hydroxy-17a-oxa-d-homo-5α-androstan-17-one (**5**)	24 (23) 71 (67)

^a^ Determined by GC analysis of the crude chloroform extracts. ^b^ Isolated yield in parentheses.

The structures of the obtained products were established by using spectroscopic techniques (IR, ^1^H-NMR and ^13^C-NMR). The assumed structures were confirmed by comparison of the characteristic shift values of selected, diagnostic signals of products and starting compounds. Resonance signals in both ^1^H-NMR and ^13^C-NMR spectra of all metabolites **5**–**8** suggested the formation of the ring-D lactones. The absence of the carbonyl group signals at δ_C_ ca. 220 ppm, the presence of new signals at δ_C_ ca. 171 ppm and downfield shifts of the C-13 resonance signals with respect to the substrates, confirmed insertion of an oxygen atom into the ring-D. The oxygen insertion into ring-D of all metabolites was supported by significant downfield shifts for both the 18-methyl protons’ signals in ^1^H-NMR and the C-18 signals in ^13^C-NMR. The bands at 1714–1720 cm^−1^ in the IR spectra of the products confirmed the δ-lactone structures. The absence of the 3α-H resonance (m) from the ^1^H-NMR spectrum of the metabolite **8** coupled with an increase in the C-19 methyl resonance signal (∆ 0.14 ppm relative to substrate **4**) and a shift of the signal of olefinic proton from δ_H_ 5.36 ppm to δ_H_ 5.75 ppm indicated isomerization of the double bond with its formation between C-4 and C-5 and oxidation of the C-3 alcohol to a ketone. Double bond migration into ring-A was fully supported in the ^13^C-NMR spectrum with downfield shifts observed for C-4 (∆ 81.8 ppm) and C-5 (∆ 28.1 ppm). Signal C-5 was visible at δ_c_ 169.1 ppm and its position was consistent with the position of a β-carbon conjugated with the carbonyl group. Oxidation of the 3β-OH group was fully supported by the loss of a methine signal at δ_c_ 71.5 ppm in the starting material ^13^C-NMR spectrum, being replaced by a new non-protonated resonance signal in the product spectrum at δ_c_ 199.4 ppm. All these observations for metabolites **5**–**8** were in agreement with those reported in the literature [6,17,18,30].

3α-Hydroxy-17a-oxa-d-homo-5α-androstan-17-one (**5**) was previously obtained in 53% yield by transformation of androsterone using *Aspergillus tamarii* KITA, but it was one of the six metabolites produced from this substrate. During that transformation, substrate as well as 3α-hydroxy-17a-oxa-d-homo-5α-androstan-17-one also underwent hydroxylation, giving a mixture of 1β- and 11β-hydroxy products [16]. Epiandrosterone was converted to 3β-hydroxy-17a-oxa-d-homo-5α-androstan-17-one (**6**) with 10% yield by *Aspergillus terreus* [18]. 17a-Oxa-d-homo-5α-androstan-3,17-dione (**7**) was previously obtained with 29% yield as one of the three metabolites by transformation of 5α-pregna-3,20-dione using *Aspergillus tamarii* KITA [11]. Testololactone (**8**) was the only product of the transformation of C_19_ or C_21_ 3-oxo-4-ene steroids or DHEA and androstenediol by *P. citreo-viride* [7], as well as progesterone by *A. terreus* [18]. This lactone was prepared with 32%–86% yield, depending on the substrate and the used biocatalyst.

**Scheme 2:**
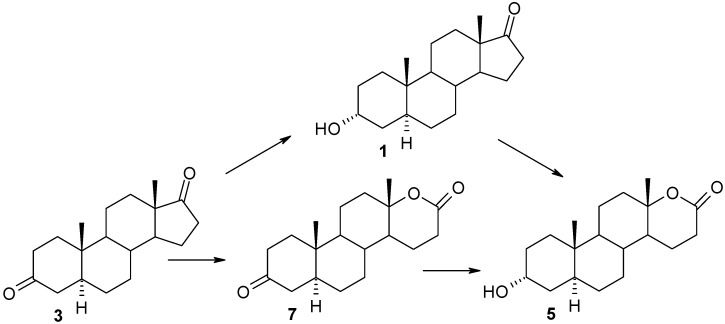
d-Lactonization pathway of androstanedione (**3**) in *P. lanosocoeruleum*.

The composition of mixtures sampled after various transformation periods was studied in order to investigate the metabolic pathways of **3** and **4** (Table 2). The reaction mixture after 8 h of incubation of androstanedione (**3**) contained, apart from the substrate, 3% of androsterone (**1**) and 2% of 3-keto-d-lactone **7**. That result indicated that substrate **3** underwent transformation via two routes: through stereoselective ketone reduction at C-3 and Baeyer-Villiger oxidation in ring-D. The rapid increase in the contents of d-lactones **5** and **7** between 8 and 12 h of the incubation indicates that compound **1** as well as compound **3** are the inducers of the BVMO responsible for the lactonization of C-17-ketones. In the product mixture obtained after 12 h and 24 h of transformation of androstanedione (**3**) the 3-oxo-lactone **7** prevailed, but its content decreased with prolonged reaction time, and consequently after 48 h of incubation, the main metabolite of **3** was 3α-hydroxy-17a-oxa-d-homo-5α-androstan-17-one (**5**). Because the amount of accumulating androsterone (**1**) did not exceed 10%, it can be assumed that lactone **5** was formed mainly from lactone **7** (Scheme 2). It was shown in a separate experiment that the 3-keto-lactone **7** was converted by *P. lanososoeruleum* to the 3α-hydroxy-lactone **5**. This is apparently the first time that the reduction of the 3-keto group has been observed in the presence of the lactone functionality in ring-d.

**Table 2:** Time course of the transformation of androstanedione (**3**) and DHEA (**4**).

Substrate	*Rt* (min)	Compounds present in the mixture (%) ^a^	Time of transformation (h)				
			4	8	12	24	48
Androstanedione (**3**)	3.75	Androstanedione (**3**)	100	95	26	5	-
	3.15	Androsterone (**1**)	-	3	8	-	-
	7.19	3α-Hydroxy-17a-oxa-d-homo-5α-androstan-17-one (**5**)	-	-	24	40	59
	7.60	17a-Oxa-d-homo-5α-androstan-3,17-dione (**7**)	-	2	40	54	39
DHEA (**4**)	3.39	DHEA (**4**)	85	26	1	-	
	4.42	Androstenedione (**9**) ^b^	15	63	58	4	
	8.42	Testololactone (**8**)	-	11	40	94	
	6.77	3β-hydroxy-17a-oxa-d-homo-androst-5-en-17-one ^c^	-	-	-	-	

^a^ Determined by GC analysis. ^b^ Identified in GC and TLC on the basis of standard. ^c^ The standard obtained in our previous work [6].

**Scheme 3:**
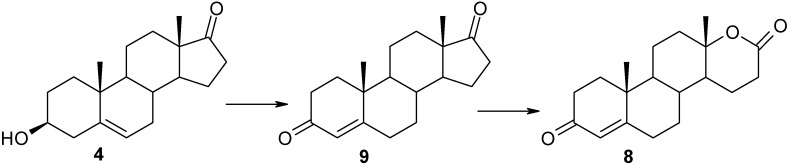
d-Lactonization pathway of DHEA (**4**) in *P. lanosocoeruleum*.

The analysis of composition of mixtures after transformation of DHEA (**4**) indicated that the first stage of the process was the oxidative conversion of 3β-hydroxy-5-ene to 3-oxo-4-ene functionality (Scheme 3, Table 2). Androstenedione (**9**) was the only metabolite identified after 4 h incubation of **4**; the mixtures after 8 h and 12 h incubation contained a significant amount of **9**. After 12 h of reaction, the content of androstenedione in the reaction mixtures started to decrease. Simultaneously, the content of testololactone (**8**) increased. The production of **8** reached a maximum at 24 h of the experiment. 3β-Hydroxy-17a-oxa-d-homo-androst-5-en-17-one was not identified in any of the reaction mixtures, which suggests that the BVMO from *P. lanosocoeruleum* does not accept 3β-hydroxy-5-ene steroids as substrates.

## 3. Experimental

### 3.1. General Conditions of Cultivation and Transformation

General experimental and fermentation details were described previously [6]. Each substrate was added to a 72-h-old culture of the microorganism as a DMSO solution, in concentration of 0.35 mmol/100 mL of medium. Each experiment was performed with four replications.

### 3.2. Chemicals and Reagents

The substrates androsterone (3α-hydroxy-5α-androstan-17-one, **1**), epiandrosterone (3β-hydroxy-5α-androstan-17-one, **2**) and dehydroepiandrosterone (3β-hydroxyandrost-5-en-17-one, DHEA, **4**) were purchased from Sigma-Aldrich (Poznań, Poland). Androstanedione (5α-androstan-3,17-dione, **3**) was obtained by oxidation of androsterone (**1**) with Jones’ reagent according to the known procedure [11] and was of high purity (>99% following GC and elemental analysis, C_19_H_28_O_2_ calcd. C, 79.12; H 9.78; found. C, 79.36; H. 9.84%. ^1^H-NMR: 0.89 (3H, s, 18-H), 1.03 (3H, s, 19-H). ^13^C NMR: 11.4 (C-19), 13.8 (C-18), 20.7 (C-11), 21.7 (C-15), 28.6 (C-6), 30.5 (C-7), 31.4 (C-12), 34.9 (C-8), 35.8 (C-16), 35.8 (C-10), 38.0 (C-2), 38.4 (C-1), 44.5 (C-4), 46.6 (C-5), 47.7 (C-13), 51.2 (C-14), 53.8 (C-9), 211.6 (C-3), 220.9 (C-17). ^1^H- and ^13^C-NMR data were found to be identical to that of androstanedione reported in the literature [31,32]. Androstenedione (**9**) was purchased from Steraloids Inc. (Newport, RI, USA). 3β-Hydroxy-17a-oxa-d-homo-androst-5-en-17-one was obtained previously in our laboratory by transformation of DHEA by *Penicillium lilacinum* AM111 [6]. The last two of the aforementioned compounds were used as analytical standards for the time course experiments. Solvents were of analytical grade.

### 3.3. Microorganism

The fungal strain *Penicillium lanosocoeruleum* KCH 3012 used in this study was taken from the collection of the Department of Chemistry, Wrocław University of Environmental and Life Sciences (Wrocław, Poland). The fungus was maintained on Sabouraud 4% dextrose-agar slopes at 4 °C and freshly subcultured before use in the transformation experiments.

### 3.4. Time Course Experiments

Time course experiments were conducted in order to determine the metabolic pathways. Conditions were identical to those in the main biotransformation experiments. One flask was harvested after 4, 8, 12 h, and every consecutive flask—after another 24 h incubation of substrate. Reaction mixtures were extracted and analyzed by GC and TLC as described above.

### 3.5. Isolation and Identification of the Products

The products of biotransformation were extracted three times with ethyl acetate. The organic extracts were dried over anhydrous magnesium sulfate, concentrated *in vacuo* and analyzed by TLC and GC. Transformation products were separated by column chromatography on silica gel with hexane/acetone (3:1 v:v) for androsterone (**1**), hexane/acetone/2-propanol (1:1:0.15 v:v:v) for epiandrosterone (**2**), hexane/acetone/ethyl acetate (1:0.6:0.3 v:v:v) for androstanedione (**3**) and hexane/acetone (1:1 v:v) for DHEA (**4**) and as eluents. TLC was carried out with Kieselgel 60 F_254_ plates (Merck, Darmstadt, Germany) using the same eluents. In order to develop the image, the plates were sprayed with solution of methanol in concentrated sulfuric acid (1:1) and heated to 120 °C for 3 min. GC analysis was performed using Hewlett Packard 5890A Series II GC instrument (FID, carrier gas H_2_ at flow rate of 2 mL min^−1^) with a HP-1 column cross-linked methyl siloxane, 30 m × 0.53 mm × 1.5 μm film thickness. The following program was used in the GC analysis: 220 °C/1 min, gradient 4 °C/min to 260° and 30 °C/min to 300°/2 min; injector and detector temperatures were 300 °C. Elemental analysis was performed on vario EL III analyzer. IR spectra were recorded in KBr disc on a Mattson IR 300 Spectrometer. The NMR spectra were measured in CDCl_3_ and recorded on a DRX 300 MHz Bruker Avance spectrometer with TMS as internal standard. Characteristic ^1^H- and ^13^C-NMR shift values in comparison to the starting compounds were used to determine structures of metabolites, in combination with DEPT analysis to identify the nature of the carbon atoms.

### 3.6. Products Isolated in the Course of Transformations

*3α-Hydroxy-17a-oxa-d-homo-5α-androstan-17-one* (**5**). C_19_H_30_O_3_, Found: C, 74.51; H, 9.86. requires C, 74.47; H, 9.87; IR ν_max_(cm^−1^): 3462, 1718. ^1^H-NMR δ (ppm): 0.73 (3H, s, 19-CH_3_), 1.28 (3H, s, 18-CH_3_), 4.05 (1H, t, *J* = 2.7 Hz, 3β-H), ^13^C-NMR δ (ppm): 172.0 (C-17), 83.4 (C-13), 66.2 (C-3), 53.0 (C-9), 46.3 (C-14), 39.3 (C-12), 38.4 (C-5), 37.9 (C-8), 36.0 (C-10), 35.5 (C-4), 31.9 (C-1), 30.5 (C-7), 28.9 (C-2), 28.6 (C-16), 28.1 (C-6), 21.5 (C-11), 20.1 (C-18), 19.6 (C-15), 11.0 (C-19). The spectroscopic data corresponded to those described in literature [17].

*3β-Hydroxy-17a-oxa-d-homo-5α-androstan-17-one* (**6**). C_19_H_30_O_3_, Found: C, 74.52; H, 9.89. requires C, 74.47; H, 9.87; IR ν_max_(cm^−1^): 3443, 1720. ^1^H-NMR δ (ppm): 0.76 (3H, s, 19-CH_3_), 1.29 (3H, s, 18-CH_3_), 3.56 (1H, m, 3α-H), ^13^C-NMR δ (ppm): 171.6 (C-17), 83.4 (C-13), 71.2 (C-3), 52.9 (C-9), 46.1 (C-14), 44.3 (C-5), 39.2 (C-12), 37.7 (C-8), 37.6 (C-4), 36.5 (C-1), 35.4 (C-10), 31.2 (C-2), 30.6 (C-7), 28.7 (C-16), 28.3 (C-6), 22.0 (C-11), 20.1 (C-18), 19.6 (C-15), 12.1 (C-19). The spectroscopic data corresponded to those described in literature [18].

*17a-Oxa-d-homo-5α-androstan-3,17-dione* (**7**). C_19_H_28_O_3_, Found: C, 74.88; H, 9.29. requires C, 74.96; H, 9.27; IR ν_max_(cm^−1^): 1721, 1714. ^1^H-NMR δ (ppm): 0.97 (3H, s, 19-CH_3_), 1.31 (3H, s, 18-CH_3_); ^13^C-NMR δ (ppm): 211.7 (C-3), 171.3 (C-17), 83.3 (C-13), 52.2 (C-9), 46.0 (C-5), 45.9 (C-14), 44.0 (C-4), 39.0 (C-12), 38.0 (C-1), 37.9 (C-2), 37.6 (C-8), 35.4 (C-10), 30.2 (C-7), 28.6 (C-16), 28.5 (C-6), 22.2 (C-11), 20.1 (C-18), 19.5 (C-15), 11.2 (C-19). The spectroscopic data corresponded to those described in literature [30].

*Testololactone (17a-oxa-d-homo-androst-4-en-3,17-dione)* (**8**). C_19_H_26_O_3_, Found: C, 75.48; H, 8.68. requires C, 75.46; H, 8.67; IR ν_max_(cm^−1^): 1717, 1664. ^1^H-NMR δ (ppm): 1.17 (3H, s, 19-CH_3_), 1.35 (3H, s, 18-CH_3_), 5.75 (1H, s, 4-H); ^13^C-NMR δ (ppm): 199.4(C-3), 171.2 (C-17), 169.1 (C-5), 124.0 (C-4); 82.7 (C-13), 52.4 (C-9), 45.9 (C-14), 39.2 (C-12), 38.4 (C-10), 38.0 (C-8), 35.3 (C-1), 33.9 (C-2), 32.4 (C-6), 30.4 (C-7), 28.7 (C-16), 30.0 (C-11), 20.0 (C-18), 19.9 (C-15), 17.45 (C-19). The spectroscopic data were in agreement with those of authentic sample isolated by us in previous studies [6].

## 4. Conclusions

The results obtained in this study indicate that the *P. lanosocoeruleum* KCH 3012 strain is a promising fungus that may be used in commercial processes, and which offers a new route to potentially active steroidal lactones. Among all the microorganisms that have been examined so far, this strain demonstrates the highest activity of Baeyer-Villiger monooxygenase against 5α-saturated steroids of the androstane series. Taking into account the catalytic abilities of this strain explored so far and its high 3β-HSD activity, one should keep in mind that it can potentially modulate the estrogen/androgen balance in Nature.
